# Polymers Used in US Household Cleaning Products: Assessment of Data Availability for Ecological Risk Assessment

**DOI:** 10.1002/ieam.4150

**Published:** 2019-07-26

**Authors:** Alison Pecquet, Drew McAvoy, Charles Pittinger, Kathleen Stanton

**Affiliations:** ^1^ Department of Environmental Health University of Cincinnati, Cincinnati Ohio USA; ^2^ Department of Chemical and Environmental Engineering University of Cincinnati, Cincinnati Ohio USA; ^3^ Charles Pittinger LLC, Cincinnati Ohio USA; ^4^ American Cleaning Institute Washington, DC USA

**Keywords:** Polymers, Cleaning products, Decision analysis, Ecotoxicology, Prioritization

## Abstract

The purpose of this study was to identify, characterize, and assess data needs for ecological risk of household cleaning product polymers currently being used in the United States (US). Because of their range in properties and functions, polymers are used in a wide variety of household cleaning products, including fabric, dish, and hard surface cleaners. Understanding their potential environmental impact is essential for good ingredient and product stewardship. The household cleaning product polymers were first identified using several databases. Of the 185 polymers initially identified, 120 were eliminated from the list because they did not fit the definition of a polymer, were not well defined (e.g., no Chemical Abstracts Service [CAS] or trade name only), or were not in current use. Forty‐seven of the remaining polymers had either adequate environmental fate and hazard data and/or sufficient data for conducting a comprehensive ecological risk assessment and were determined to be of low concern by either the United States Environmental Protection Agency (USEPA), the European Chemicals Agency (ECHA), and/or the Human and Environmental Risk Assessment (HERA) Project. The remaining 18 polymers were determined to need further review because of a lack of publicly available information for conducting ecological risk assessments. Additional data for these 18 polymers could be obtained by accessing privately held data, conducting laboratory tests on their fate and effects in aquatic environments, or by conducting read‐across of similar structured polymers. These steps can be utilized by industry to determine where best to dedicate future environmental stewardship efforts. *Integr Environ Assess Manag* 2019;15:621–632. © 2019 The Authors. *Integrated Environmental Assessment and Management* published by Wiley Periodicals, Inc. on behalf of Society of Environmental Toxicology & Chemistry (SETAC)

## Key Points

- This project aimed to identify, characterize, and obtain environmental information on United States (US) household cleaning product polymers in current use to identify polymers that need additional data for conducting ecological risk assessments.
- An initial list of 185 polymers was narrowed by excluding polymers that were adequately characterized for ecological risk and those that did not fall under the Organisation for Economic Co‐operation and Development (OECD) definition of a polymer.
- A comprehensive literature search and compilation of publicly available data allowed for conducting a data gap analysis of the remaining 18 polymers, although little data were available.
- Further study to inform risk assessments for these polymers could include QSAR modeling, read‐across, accessing privately held data, or further testing.

## INTRODUCTION

The cleaning products industry has a long‐standing history of environmental stewardship for their product ingredients. Most of their research has focused on surfactants and chelating agents used in household cleaning products. Recently, regulatory authorities and nongovernmental organizations (NGOs) have focused their attention on the fate and potential effects of polymers in the environment, and in particular, plastic fibers and particles. Because of this interest in polymers, a study was initiated by the American Cleaning Institute (ACI) to assess what is known about polymers currently being used in household cleaning products marketed in the United States (US).

Polymers represent a large class of organic compounds that consist of repeating monomer units connected by covalent bonds (OECD 1990; Sperling 2006; ECHA 2012; Ravve 2012). Polymers are classified as soluble or insoluble, with soluble polymers further classified as nonionic, cationic, anionic, and amphoteric compounds. Key factors in determining the properties of polymers are monomer composition, polymer chain length, and molecular weight (MW) distribution. The physical properties of a polymer (e.g., viscosity, shear stress, adhesion, and flocculation) dictate their function (e.g., emulsifier, solubility enhancer, viscosity modifier, dispersant, or defoamer) in cleaning product formulations. These properties also determine their fate and ecological effects in the environment, thus data on physical properties can be utilized in predicting potential ecological toxicity.

Due to knowledge gained over time, the United States Environmental Protection Agency (USEPA) regulatory scientists have developed general principles or criteria based on physical–chemical properties for determining “polymers of low concern” (USEPA 1997, 2013). Criteria for polymers of low concern have also been developed by the Organisation for Economic Co‐operation and Development (OECD) Expert Group on polymers (OECD 1990, 1993), which subsequently concluded that “Polymers of low concern are those deemed to have insignificant environmental and human health impacts” (OECD 2009). These principles involve an assessment of the polymer's composition, MW and distribution, ionic character, functional groups, low MW leachable compounds, particle size, structural and elemental composition, water and lipid solubility, and stability (abiotic, biotic, and thermal). In general, polymers with MW >1000 Da have been regarded as having inherently low toxicity due to their inability to cross cell membranes (Boethling and Nabholz 1996), whereas polymers with an average MW (M_n_) <1000 Da and an oligomer content >1% are considered a potential health concern (OECD 2009). Also, larger polymers that degrade into smaller molecules could have the potential to cross cell membranes and thus potentially exhibit effects to humans and the environment. Cationic polymers are also considered compounds of concern with regard to aquatic toxicity (Landis et al. 1993; USEPA 1997), and according to the Stockholm Convention, polymers with an octanol–water partition coefficient (*K*_ow_) <5000 are considered to have a low potential to bioaccumulate (Henry et al. 2018). The principles for determining polymers of concern for several countries are provided in the Supplemental Data.

Across the universe of polymer chemistries, the cleaning product industry uses a narrow and selective fraction of polymers. Most of the polymers used in down‐the‐drain household cleaning products (e.g., dish soaps, laundry detergents, fabric softeners, and hard surface cleaners) are water soluble. Thus, the focus of the present study was to identify, characterize, and assess environmental fate and effects data gaps for soluble household cleaning product polymers currently being used in the USA. This investigation had 5 tasks:

1) identify polymers currently used in US household cleaning products, including fabric, dish care, and hard surface cleaners;
2) verify that the polymers meet the US definition of a polymer and are in current use;
3) characterize the polymers by their chemical composition, physical–chemical properties, and function in product formulations;
4) compile publicly available environmental fate and ecological effects data; and
5) identify data gaps for ecological risk assessments.

**Figure 1:**
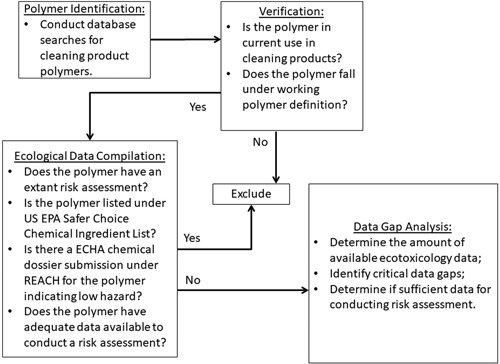
Identification of polymers and available data for US cleaning products. Polymers were first identified through wide searches in multiple databases, then verified as polymers and if in current use. Polymers were excluded if previous risk assessments were available or sufficient data were available to conduct a risk assessment. Remaining polymers were subject to data gap analysis and prioritization. ECHA = European Chemicals Agency; USEPA = United States Environmental Protection Agency.

These tasks were achieved by conducting database searches, an extensive literature review, and consultation with technical experts in the household cleaning product industry. A schematic of the approach taken is provided in Figure 1. Although the focus of this study was on US household cleaning product polymers in current use, the approach taken could also be used by any sector of the chemical industry to identify future environmental stewardship efforts.

## IDENTIFICATION OF POLYMERS CURRENTLY USED IN US CLEANING PRODUCTS

Cleaning product polymers used in the USA were first identified by conducting database searches. Five databases were searched: the Consumer Product Information Database (CPID 2018), the Household Products Database (HPD; NLM 2017), the Ecology Product Testing Database (EPTD; WDoE 2014), the Human and Environmental Risk Assessment on Ingredients of Household Cleaning Products Database (HERA 2018), and the American Cleaning Institute's Cleaning Product Ingredient Safety Initiative (CPISI) Inventory (ACI 2012). Additionally, the PubMed database (2019) was searched for general information on polymers used in cleaning products, and for broad information on polymer chemistries and classification. The CPID search for the term “polymer” returned 3 consumer products (none were cleaning products) and 299 chemicals. Similarly, there were 2777 hits when searching “cleaners” in the HPD. The EPTD had to be searched either by specific chemical or by specific product and therefore was not useful for polymer mining. In order to determine the relevance of the cleaning products and polymers identified in these 3 databases, an evaluation of each product formulation should be conducted to see if the polymers are being used in cleaning products. However, many times these data are either proprietary or the polymers are inadequately characterized. Due to these limitations, this extensive analysis of multiple databases and individual cleaning products was beyond the scope of the present project.

From the database searches, 185 prospective polymers were initially identified with the majority coming from the ACI CPISI database. These data were supplemented with polymers identified in the HERA assessments (HERA 2018).

The PubMed database was preliminarily searched for information on polymer chemistries for use in cleaning products:

- Polymer classification (returned 603 819 results)
- Cleaning product AND polymer (returned 134 539 results)
- Cleaning product polymer chemistry (returned 75 130 results)
- Polymer chemistries for cleaning products (returned 74 982 results)
- Polymer AND consumer product NOT plastic (returned 287 results)
- Polymer AND consumer product NOT plastic NOT cosmetic (returned 241 results).

The majority of hits from the broad electronic library search were not relevant. The categories were either too broad, as in the case of the HPD, too narrow, as in the CPID search, and/or the results included nonrelevant data such as information on plastics (majority of information), information on manufacturing, and information on synthesis and molecular chemistries. Several articles on polymers in consumer products were identified, but most referred to hard plastics and marine pollution. The limited relevant citations were saved.

Information resulting from the searches on hard plastics, microplastics, plasticizers, and manufacturing and engineering processes were excluded from the search results because these were beyond the scope of the present project. In addition, silicone‐based polymers (e.g., methylcyclotetrasiloxane) were excluded from the search because they are being evaluated by the Global Silicone Council (GSC 2015). Major polymeric surfactants in common use were removed from the list because they have been extensively evaluated for their safety (Cowan‐Ellsberry et al. 2014). Personal care products (PCPs) such as bathing soaps and shampoos were excluded from the search because these products were out of scope for the ACI, with the caveat that some polymers are used in both PCPs and cleaning products. Polymers identified only by trade names and those lacking CAS numbers were removed from the list because of inadequate characterization. Because water solubility can often decrease with increasing polymer chain length (Ravve 2012), high‐MW polymers (>10 000 Da), thermoset polymers (irreversibly hardened into an insoluble polymer network), and water‐absorbing polymers that form gels in situ were also excluded from the list.

Finally, compounds that did not conform to the US–OECD polymer definition and criteria (OECD 1990) were removed, which chiefly included enzymes and colorants. The details of the OECD polymer definition are evaluated alongside other regulatory definitions and are provided in the Supplemental Data. The OECD polymer definition was agreed upon in May 1993 by OECD member countries, including the United States, Canada, China, Korea, Japan, and member nations of the European Union. The OECD definition and, therefore, the US definition of a polymer is:

A “POLYMER” is a substance consisting of molecules characterized by the sequence of one or more types of monomer units and comprising a simple weight majority of molecules containing at least three monomer units which are covalently bound to at least one other monomer unit or other reactant and consists of less than a simple weight majority of molecules of the same molecular weight. Such molecules must be distributed over a range of molecular weights wherein differences in the molecular weight are primarily attributable to differences in the number of monomer units (OECD 2019).

For deciding whether a substance meets the definition of a polymer, the following criteria must be met:

- More than 50% of molecules must be composed of a sequence of at least 3 monomer units plus at least 1 additional monomer unit or other reactant. In other words, >50% of the substance must be polymer molecules.
- The amount of polymer molecules of any 1 MW cannot exceed 50% by weight.

By this definition, many low‐MW substances, oligomeric reaction products, dimers, or trimers would not be classified as a polymer.

Because product formulators may replace product ingredients with better performing and/or more cost‐effective compounds, a commercial use survey was conducted for the 185 prospective polymers identified in the database searching. Eight ACI member companies representing manufacturers and formulators were surveyed to confirm the identified polymers are in current use, and to identify polymers in current use that were not identified on the list. From this survey, 60 of the listed polymers were confirmed to be in use and 5 additional polymers not on the original list were identified.

Following this evaluation, of the 185 prospective polymers identified, 120 of the original polymers were removed from the list. The final list of polymers verified by the industry survey totaled 65 polymers (Tables 1a and 1b).

**Table 1a:** Household cleaning product polymers (fabric, dish, and hard surface cleaners) used in 2016 with safety assessments conducted by the USEPA, the ECHA, and/or the HERA program and the assessment conclusion

Polymer classification	Polymer name	CAS nr	USEPA, ECHA, and HERA assessments and assessment conclusion^a^
Polyacrylate	Polyacrylic acid homopolymers and their sodium salts; generic CAS nr	9003‐04‐7	USEPA: Green circle
			HERA: does not pose a risk to the environment
	2‐Propenoic acid, homopolymer	9003‐01‐4	USEPA: Green circle
			HERA: does not pose a risk to the environment
	2‐Propenoic acid, sodium salt, homopolymer	25549‐84‐2	HERA: Does not pose a risk to the environment
	2‐Propenoic acid, homopolymer, sodium salt, isotactic	28603‐11‐4	HERA: Does not pose a risk to the environment
	2‐Propenoic acid, telomere with sodium hydrogen sulfite, sodium salt	68479‐09‐4	USEPA: Green circle
			HERA: does not pose a risk to the environment
	Acrylate copolymer	25035‐69‐2	USEPA: Green circle
	2‐Propenoic acid, 2‐methyl‐, polymers with Et acrylate and polyethylene glycol methacrylate C16‐18‐alkyl ethers	70879‐60‐6	USEPA: Green circle
	Acrylate copolymer	25212‐88‐8	USEPA: Green circle
MA‐acrylate copolymer	Polyacrylic/maleic acid copolymers and their sodium salts: Generic CAS nr	52255‐49‐9	USEPA: Green circle
			HERA: does not pose a risk to the environment
	2‐Butenedioic acid (Z), polymer with 2‐propenoic acid	29132‐58‐9	USEPA: Green circle
			HERA: does not pose a risk to the environment
	2‐Butenedioic acid (Z), monosodium salt, polymer with sodium 2‐propenoate	51025‐75‐3	HERA: Does not pose a risk to the environment
	2‐Butenedioic acid (Z), sodium salt, polymer with sodium 2‐propenoate	51344‐35‐5	HERA: Does not pose a risk to the environment
	2‐Butenedioic acid, disodium salt, polymer with sodium 2‐propenoate	60449‐78‐7	HERA: Does not pose a risk to the environment
	2‐Butenedioic acid (Z), polymer with 2‐propenoic acid, sodium salt	60472‐42‐6	HERA: Does not pose a risk to the environment
	2‐Butenedioic acid (Z), disodium salt, polymer with 2‐propenoic acid	61842‐61‐3	HERA: Does not pose a risk to the environment
	2‐Butenedioic acid (Z), monosodium salt, polymer with 2‐propenoic acid	61842‐65‐7	HERA: Does not pose a risk to the environment
	2‐Butenedioic acid (Z), sodium salt, polymer with 2‐propenoic acid	63519‐67‐5	HERA: Does not pose a risk to the environment
	2‐Butenedioic acid (Z), disodium salt, polymer with sodium 2‐propenoate	112909‐09‐8	HERA: Does not pose a risk to the environment
	2‐Butenedioic acid (Z), polymer with sodium 2‐propenoate	126595‐54‐8	HERA: Does not pose a risk to the environment
Polyolefin	Polybutene	9003‐29‐6 9003‐28‐5	USEPA: Green circle
			ECHA: data conclusive for low hazard
Cellulose ether	Cellulose gum (sodium carboxymethyl cellulose)	9004‐32‐4	USEPA: Green circle
	Methylcellulose	9004‐67‐5	USEPA: Green circle
	Hydroxyethylcellulose	9004‐62‐0	USEPA: Green circle
	Hydroxypropyl methylcellulose	9004‐65‐3	USEPA: Green circle
	Starch	9005‐25‐8	USEPA: Green circle
	Xanthan gum	11138‐66‐2	USEPA: Green circle
Polyether	Polyethylene‐polypropylene glycol	9003‐11‐6	USEPA: Green circle
	Glycereth – 26	31694‐55‐0	USEPA: Green circle
			ECHA: Data conclusive for low hazard
	Polyethylene glycol	25322‐68‐3	USEPA: Green half‐circle
			ECHA: Data conclusive for low hazard
	Polyethylene glycol	112‐60‐7	USEPA: Green half‐circle
			ECHA: Data are sufficient for low hazard
	Polypropylene glycol	25322‐69‐4	USEPA: Yellow triangle
			ECHA: Data conclusive for low hazard
	Buteth‐3	9004‐77‐7	ECHA: Data conclusive for low hazard
	Polyethylene glycol distearate	9005‐08‐7	USEPA: Green circle
	Polyethylene glycol isotridecyl ether	127036‐24‐2	USEPA: Green circle
Ethoxylated triglyceride	PEG castor oil	61791‐12‐6	USEPA: Green circle
	PEG‐40 hydrogenated castor oil	61788‐85‐0	ECHA: Data conclusive for low hazard
MA/olefin copolymer	Sodium MA/diisobutylene copolymer	37199‐81‐8	USEPA: Green circle
Styrene/acrylate copolymer	Styrene/acrylates copolymer	9010‐92‐8	USEPA: Green circle
Anionic polysaccharide	Sodium carboxymethyl inulin	430439‐54‐6	USEPA: Green circle
Polyol	Polyvinyl alcohol	9002‐89‐5	USEPA: Green circle
	Polyvinyl acetate ‐ Polyvinyl alcohol	25213‐24‐5	USEPA: Green circle
	Ethoxylated coco alkylbis(hydroxyethyl) methyl ammonium chloride	61791‐10‐4	USEPA: Green circle
Ethoxylated sorbitan ester	Polysorbate 20	9005‐64‐5	USEPA: Green circle
			ECHA: Data conclusive for low hazard
	Polysorbate 80	9005‐65‐6	USEPA: Green circle
	Polysorbate 65	9005‐71‐4	USEPA: Green half circle
Alkylether sulfosuccinate ester	Disodium laureth sulfosuccinate	68815‐56‐5	USEPA: Green circle
Polyvinyllactam	Polyvinyl pyrolidone	9003‐39‐8	USEPA: Green circle

CAS = Chemical Abstracts Service; ECHA = European Chemicals Agency; HERA = Human and Ecological Risk Assessment program; USEPA = US Environmental Protection Agency.

^a^ Green circle: the chemical has been verified to be of low concern based on experimental and modeled data; Green half‐circle: the chemical is expected to be of low concern based on experimental and modeled data; Yellow triangle: chemical has met Safer Choice Criteria for its functional ingredient class but has some hazard profile issues.

**Table 1b:** Household cleaning product polymers (fabric, dish, and hard surface cleaners) used in 2016 without published safety assessments from the USEPA, the ECHA, or HERA

Polymer classification	Polymer name	CAS nr	Function in cleaning products
Polyacrylate	Acrylate copolymer	25133‐97‐5	Opacifier, viscosity modifier
	P(AA/EA/MAA)	30351‐73‐6	Antideposition
Polycarboxylate	Styrene/acrylates copolymer	25034‐86‐0	Opacifier, viscosity modifier
	Styrene/acrylates copolymer	25085‐34‐1	Opacifier, viscosity modifier
Acrylate/sulfonate copolymer	AA/AMPS copolymer	40623‐75‐4	Dispersing agent, antideposition
Styrene/MA copolymer	Styrene/MA copolymer	9011‐13‐6	Dispersing agent
Styrene/acrylamide copolymer	Styrene/acrylamide copolymer	24981‐13‐3	Opacifier, viscosity modifier
Ethoxylated Polyethyleneimine	Polyethyleneimine ethoxylate	68130‐99‐4	Dispersing agent, emulsifier
Polyester	Polyester‐5	54590‐72‐6	Dispersing agent
Polyglyceryl ester	Polyglyceryl oleate	9007‐48‐1	Emulsifier, dispersing agent
Polyether	Ethoxylate alcohols, lauryl	3055‐97‐8	Surfactant
Quaternary ammonium	Polyquaternium‐5	26006‐22‐4	Flocculant
	Polyquaternium‐6	26062‐79‐3	Surface modifier, flocculant
	Polyquaternium‐7	26590‐05‐6	Surface modifier
	Polyquaternium‐2	68555‐36‐2	Conditioning agent
	Polyquaternium‐10	68610‐92‐4	Conditioning agent
Resin	Melamine resin	9003‐08‐1	Film former, antiwrinkle
Starch alkenylsuccinate ester	Sodium starch octenyl succinate	52906‐93‐1	Emulsifier, viscosity modifier

AA/AMPS = 2‐acrylamido‐2‐methylpropanesulfonic acid‐acrylic acid copolymer; CAS = Chemical Abstracts Service; ECHA = European Chemicals Agency; HERA = Human and Ecological Risk Assessment program; MA = maleic anhydride; P(AA/EA/MAA) = acrylic acid, ethyl prop‐2‐enoate, 2‐methylprop‐2‐enoic acid; USEPA = US Environmental Protection Agency.

## POLYMER RISK ASSESSMENTS AND DATA GAP ANALYSIS

A key goal of the present project was to assess data gaps for polymers in current use. The first step was to screen polymers for the availability of published ecological risk assessments. These assessments were used to confirmed whether sufficient data are available to conduct an assessment, and to determine whether the polymer is considered “safe” (e.g., polymer of low concern) by conventional criteria. Polymers that lacked sufficient data for conducting an ecological risk assessment were assigned to the subsequent data gap analysis. In the present evaluation ecological risk assessments were compiled from databases of 2 regulatory agencies: ECHA (ECHA 2018) and the USEPA (USEPA 2018a, 2018b). Assessments for the polyacrylate and polycarboxylate polymers were also compiled from 2 publications by the HERA industry‐sponsored research program (HERA 2014a, 2014b).

The polymers in current use were searched by CAS number in the ECHA Information on Chemicals database (ECHA 2018) to identify those with dossiers submitted under the EU's Registration, Evaluation, Authorisation and Restriction of Chemicals (REACH) (2016) program. Dossiers for 8 of the polymers were identified (Table 1a), which were evaluated for data and summaries regarding hazard classification by ECHA. It should also be noted that 6 of the 8 ECHA polymers were evaluated under USEPA's program and 5 were assigned “green circle” (see Table 1a). Subsequently, those polymers with adequate data and/or hazard labeling conclusions for low ecological risk and ecotoxicological hazard (8 polymers) were removed from the list (Table 1a).

In addition, polymers in current use were also compared to the USEPA Safer Choice Chemical Ingredient List (USEPA 2018a). Thirty‐two of the polymers were either coded as “green circles,” indicating the chemical has been verified to be of low concern based on experimental and modeled data, or “green half‐circle,” meaning the chemical is expected to be of low concern based on experimental and modeled data. Polypropylene glycol is listed as “yellow triangle,” which is defined by the USEPA as having “met Safer Choice Criteria for its functional ingredient‐class but has some hazard profile issues.” This classification is likely due its volatility as a solvent, though it is VOC‐exempt under California Air Resources Board and USEPA criteria. However, for this polymer, the ECHA dossier presents fate and transport data that show it is readily biodegradable, has a low potential for bioaccumulation, and has a low affinity for adsorption to soils and activated sludge biosolids. Additional data in the ECHA assessment for ecotoxicology show it does not warrant classification according to the Globally Harmonized System of Classification and Labelling of Chemicals (GHS) and the substance is not persistent, bioaccumulative, or toxic (PBT). Therefore, this polymer and the other polymers marked with green circles or green half circles were removed from the final list (Table 1a).

Polyacrylate polymers in current use were cross‐referenced with environmental risk assessments conducted by HERA (2018). The HERA program is a voluntary industry initiative launched in 1999 by the International Association for Soaps, Detergents and Maintenance Products (AISE; representing cleaning product formulators and manufacturers) and the European Chemical Industry Council (Cefic; representing ingredient suppliers and manufacturers of the raw materials). Polycarboxylate polymers, including polyacrylic acid homopolymers (HERA 2014a) and polyacrylic and maleic acid copolymers (HERA 2014b), represent the largest chemical class of polymers used in US cleaning products. The resulting conclusions from the HERA assessments is that there is no indication of “environmental risks for all relevant compartments including water, sediment, soil, and sewage treatment plant (STP) with all risk characterization ratios (RCRs) below 1. The outcome of this present environmental risk assessment provides a sound basis for the conclusion that the use of homopolymers in detergent products does not pose a risk to the environment.” This conclusion is applied to 16 polycarboxylates currently being used in cleaning products that were evaluated by the HERA program (Table 1a).

Of the 65 polymers in current use, 47 were found to have ecological assessments by at least 1 of the 3 sources (HERA, USEPA, ECHA), with some of the polymers having assessments from more than 1 organization (Table 1a). The remaining 18 polymers lacked assessments from any source and were targeted for further data compilation.

## ENVIRONMENTAL FATE AND EFFECTS DATA COMPILATION

Electronic literature and database searches were conducted for the remaining 18 nonassessed polymers to compile data on their ecological fate (sorption, biodegradation, and bioaccumulation) and effects (aquatic acute and chronic toxicity). The searches used 4 broad chemical databases: Scopus (2019), Web of Science (2019), Environment Complete (2019; including Academic Search Complete, Academic Search Premier, and Agricola), and Toxline (2019; including PubMed). All searches were conducted from September to November 2016. These databases were searched first by CAS number. If there were no hits, then the chemical name as listed in the polymer inventory was used. The USEPA ECOTOX database (USEPA 2017) was also searched for safety data using the chemical CAS number. A summary of the searches can be found in the Supplemental Data, including the exact search strings and total number of hits for each chemical for each database.

Once studies were determined to be potentially relevant, they were downloaded as titles and abstracts from each database. The studies were grouped by chemical category in an attempt to minimize the number of duplicate studies across polymers. The abstracts and titles were then reviewed for relevance. Once an abstract was deemed relevant to the present research, the full manuscript was retrieved to determine if there were any data that could be used in an ecological risk assessment.

Limited data were found on the fate (sorption, biodegradation, bioaccumulation, and removal) and ecotoxicity (invertebrate, fish, and algae) of the remaining 18 identified polymers. A majority of the identified data was for quaternary ammonium compounds. These data included algal, fish, and invertebrate acute ecotoxicity and chronic fish ecotoxicity (Table 2a). Only 3 other polymers had acute invertebrate ecotoxicity data. Additional data for the quaternary ammonium compounds (polyquats) included fate in wastewater treatment plants (Table 2a). None of the remaining polymers had any data on environmental fate and transport. The data from these sources were compiled to identify data gaps for conducting future risk assessments (Tables 2a and 2b). However, minimal data on ecotoxicity or fate and effects in the environment were identified for most of the listed polymers, and these data gaps are discussed in the *Data Gap Analysis* section.

**Table 2a:** Publicly available fate data for polyquat cleaning product polymers that do not have published assessments from the USEPA, the ECHA, and/or the HERA program

Estimated wastewater treatment plant removals									
Polymer classification	Name	CAS nr	Variant	Charge density^a^	Avg MW (kDaL)^b^	% N^b^	Viscosity (as 2% aq sol’n) (mPa/s)^b^	*K*_d_ (L/g)^c^	Estimated % WWTP^d^
Quaternary ammonium	Polyquaternium‐6	26062‐79‐3	Magnafloc 368, Veligon	6.2	50	ND	ND	2200 (humic acid)	38
	Polyquaternium‐10	68610‐92‐4	UCARE JR125	0.9	250	1.5–2.2	75–125	470 (humic acid)	13–16
			UCARE JR30M	1.0	600	1.5–2.2	30 000	630 (humic acid)	13–16
			UCARE JR400	1.2	400	1.5–2.2	300–500	440 (humic acid)	13–16
			UCARE LK	0.3	Low	0.4–0.6	300–500	Not available	13–16
			UCARE LR30M	0.4	30	0.8–1.1	30 000	Not available	13–16
			UCARE LR400	0.6	Low	0.8–1.1	300–500	Not available	13–16

CAS = Chemical Abstracts Service; ECHA = European Chemicals Agency; HERA = Human and Ecological Risk Assessment; MW = molecular weight; ND = no data; USEPA = US Environmental Protection Agency; WWTP = wastewater treatment plant.

^a^ Source: Cumming 2008.

^b^ Source: Cumming et al. 2008.

^c^ Source: Cumming et al. 2010.

^d^ Source: Cumming et al. 2011a.

**Table 2b:** Publicly available effects data for polyquat, ether, resin, and styrene cleaning product polymers that do not have published assessments from the USEPA, the ECHA, and/or the HERA program

Fish acute toxicity data				
Polymer classification	Name	CAS nr	Variant	96‐h acute fish (mg/L)
Quaternary ammonium	Polyquaternium‐6	26062‐79‐3	Magnafloc 368, Veligon	EC50 = 0.5 (*Gambusia holbrooki*)^a^
			Unknown	LC50 = 0.22 (*Pimephales promelas*)^b^
			Unknown	LC50 = 0.26 (*P. promelas*)^b^
			Unknown	NOEL = 0.11 (*P. promelas*)^b^
			Unknown	NOEL ≤ 0.10 (*P. promelas*)^b^
			Unknown	LC50 = 0.066 (*Oncorhynchus mykiss*)^b^
			Unknown	LC50 = 0.077 (*O. mykiss*)^b^
			Unknown	NOEL = 0.043 (*O. mykiss*)^b^
			Unknown	NOEL ≤ 0.059 (*O. mykiss*)^b^
			Magnafloc 368	LC50 = 2.08 (*Salvelinus namaycush*)^c^
	Polyquaternium‐10	68610‐92‐4	UCARE JR125	EC50/LC50 = 1.2 (*G. holbrooki*)^a^
			UCARE JR30M	EC50/LC50 = 1.5 (*G. holibrooki*)^a^
			UCARE JR400	EC50/LC50 = 2.1 (*G. holbrooki*)^a^
			UCARE LK	EC50/LC50 = 100 (*G. holbrooki*)^a^
			UCARE LR30M	EC50/LC50 = 66 (*G. holbrooki*)^a^
			UCARE LR400	EC50/LC50 = 64 (*G. holbrooki*)^a^

Invertebrate acute toxicity data				
Polymer classification	Name	CAS nr	Variant	Acute toxicity data (mg/L)
Ether	Ethoxylated alcohols, lauryl	3055‐97‐8	NA	EC50 = 2.2 (*Daphnia magna*)^b^
				EC50 = 0.52 (Ciliate)^b^
Quaternary ammonium	Polyquaternium‐6	26062‐79‐3	NA	EC50 = 0.075 mg/L (*D. magna*, immobile)^b^
				EC50 = 2.1 mg/L (*D. magna*, immobile)^b^
				EC50 = 0.51 mg/L (*Ceriodaphnia*, mortality)^b^
				EC50 = 0.54 mg/L (*Ceriodaphnia*, mortality)^b^
				EC50 = 0.32 mg/L (*Ceriodaphnia*, mortality)^b^
				EC50 = 0.77 mg/L (*Ceriodaphnia*, mortality)^b^
				NOEL = 0.50 mg/L (*D. magna*, immobile)^b^
				NOEL ≤ 0.059 mg/L (*D. magna,* immobile)^b^
Resin	Melamine resin	9003‐08‐1	NA	LC50 = 10.9 (*D. magna*)^b^
Styrene	Styrene/MA copolymer	9011‐13‐6	NA	EC50 = 10.0 (*D. magna,* mortality)^b^

Algae acute toxicity data				
Polymer classification	Name	CAS nr	Variant	Algae 72 h (*Chlorella sp12*) (mg/L)^d^
Quaternary ammonium	Polyquaternium‐6	26062‐79‐3	Magnafloc 368, Veligon	EC50 = 0.03
	Polyquaternium‐10	68610‐92‐4	UCARE JR30M	EC10 = 0.002
				EC50 = 0.05
			UCARE JR125	EC50 = 0.04
			UCARE JR400	EC10 = 0.013
				EC50 = 0.05

Chronic toxicity data				
Polymer classification	Name	CAS nr	30‐d fish (*S. namaycush*) (mg/L)^c^	7‐d invertebrate (mg/L)
Quaternary ammonium	Polyquaternium‐6	26062‐79‐3	NOEC = 0.5, survival and growth	EC20 = 0.0042 (*C. dubia*, reproduction)^b^
			LOEC = 1.0, survival and growth	EC50 = 0.14 (*C. dubia*)^b^
				NOEL ≤ 0.059 (*D. magna*)^b^

CAS = Chemical Abstracts Service; ECHA = European Chemicals Agency; HERA = Human and Ecological Risk Assessment Program; LOEC = lowest observed effect concentration; NA = not available; NOEC = no observed effect concentration; NOEL = no observed effect level; USEPA = US Environmental Protection Agency.

^a^ Source: Cumming et al. 2011b.

^b^ Source: USEPA 2017.

^c^ Source: Liber et al. 2005

^d^ Source: Cumming 2008.

## DATA GAP ANALYSIS

Data gaps are evident for the 18 identified polymers with some published data to support ecological risk assessments for 5 of the polymers. The remaining 13 polymers had no reported data. A summary of the data gap assessment is provided in Table 3, and general conclusions on polymer class are presented below.

**Table 3:** Data gap assessment and summary by polymer class

Polymer classification	Apparent data gaps by chemical class
Acrylate	Ethyl acrylate (1 compound). No data found for fate or toxicity. Further investigation recommended.
Carbohydrate	Butanedioate (1 compound). No data identified. Likely readily biodegradable and low toxicity.
Ester	Ester (2 compounds). No data found for fate or toxicity. Further investigation recommended.
Ether	Ether (1 compound). Further chemical characterization needed.
Polycarboxylate	Polycarboxylates not reviewed by HERA or USEPA (4 compounds). This is a broad chemical class, and the polymers identified require better chemical characterization. There is likely good read‐across potential to data from compounds reviewed by HERA.
Polyethyleneimine	Polyethyleneimine (1 compound). No data found for fate or toxicity. Further investigation recommended.
Quaternary Ammonium Compound	Quaternary ammonium cationic polymer (5 compounds). Polyquats 6 and 10 have published data. Polyquat 6 was assessed by ECCC/HC as part of a poly(amine)s group. No data available on other polyquats.
Resin	Melamine resin (1 compound). No fate data and limited toxicity data. Further investigation recommended.
Styrene	Styrene (2 compounds). No fate and limited toxicity data found. Further investigation recommended.

ECCC/HC = Environment and Climate Change Canada Health Canada; HERA = Human and Ecological Risk Assessment Program; USEPA = United States Environmental Protection Agency.

Polycarboxylates and polyacrylates represent a significant use category by the cleaning products industry, with high economic value and indispensable properties in many cleaning applications. Although the environmental fate and effects of polycarboxylates and polyacrylates have been extensively studied (HERA 2014a, 2014b), environmental information for some of the polycarboxylates used in cleaning products but not included in the HERA report is limited. Four polycarboxylates were identified as needing additional data for conducting ecological risk assessments, although, there is a high read‐across potential using data from the polycarboxylates reviewed by the HERA program.

The USEPA has a special caution for cationic polymers, such as the quaternary ammonium compounds, due to their potential toxicity. Among the 5 polyquats, only polyquats 6 and 10 have published data (Tables 2a and 2b). For the remaining polyquats, additional investigation is warranted. For other polymers, such as carbohydrates, a long history of safe use may reduce the priority for further investigation. Other categories (resins, esters, and polyethyleneimines) had no data identified and therefore further investigation is warranted. For the remaining polymer categories (ethers, ethyl acrylates, and styrenes), some data are available and there is a high potential for possible read‐across from other compounds. Additionally, the long history of safe use could indicate potential for low ecotoxicity. However, additional research is needed to determine the sufficiency of these approaches.

Overall, very little data were available for the 18 remaining polymers. The relative paucity of data for these polymers was not unexpected for several reasons:

1) Polymers were removed from the initial list if they had previously been reviewed in some depth (e.g., by HERA, the USEPA, or ECHA). Polymers with extant assessments were more data rich compared to the 18 polymers on the final list, hence enabling their previous assessments.
2) Polymers in general have historically received less attention than lower MW chemicals, due to the observation that larger (e.g., >1000 Da) molecules are impeded from crossing cell membranes. A great deal of information is required to adequately characterize polymers on the basis of MW and size distribution. However, this type of data was not available for a large proportion of the identified polymers.
3) Polymers tend to exhibit lower toxicity in conventional ecotoxicity tests, except for the polyquats discussed previously. However, polymers with low water solubility could induce physical effects if tested above their water solubility. More standardized testing protocols for physical effects of insoluble polymers should be a research priority.

## FUTURE STUDY

The present project built a strong foundation for cleaning product manufacturers to evaluate polymers used in US household cleaning products. A number of key next steps for further study include the following:

1) characterization of the remaining 18 identified polymers, for example, chain length, percent monomers, percent byproducts, and structural changes during formulation and use that may alter solubility;
2) prioritization of the identified polymers based on broader criteria that include economic importance to the industry, breadth of use in products, down‐the‐drain use volume, and concentration in product, which can further inform priorities and decisions for further investigations of polymers;
3) refinement of the data gaps by conducting read‐across analyses with other polymers in the same chemical class;
4) determination of physical, chemical, and biological data gaps that could be filled cost effectively and then ranked by polymers of greatest potential concern; and
5) evaluation of current risk assessment models for their ability to predict ecological risk of polymers and conduct a sensitivity analysis to determine the most important environmental fate parameters for predicting ecological risk.

## CONCLUSIONS

The present effort was undertaken to better understand the landscape of polymers used in household cleaning products and to identify data gaps for conducting ecological risk assessments. The approach taken in the present study can serve as a blueprint for other industries to determine where stewardship efforts should be taken and to ensure that further investigative resources are focused on priority data gap areas. This approach allowed us to refine an initially large list of possible polymers (185) to a relatively narrow list of polymers for future investigation (18). Further study to inform risk assessments for these polymers could include QSAR modeling, read‐across, accessing privately held data, or further testing.

Finally, it should be emphasized that the results of the present study (i.e., the remaining 18 polymers) are not indicative of ecological risk. They merely indicate the need for collecting additional fate and effects data that would be required to perform ecological risk assessments. It was beyond the scope of the present project to assess the risk of these polymers. In particular, ecological risk assessments would require an estimation of exposure concentrations, which were not determined in the present study. The polymers identified offer a path forward for future data generation to inform ecological risk assessments.

## Data Accessibility

Data are accessible in the Supplemental Data files.

## SUPPLEMENTAL DATA

Summary data on the criteria used to screen polymers, and detailed information on literature searching and results.

## Supporting information

This article includes online‐only Supplemental Data.

Supporting information
