# Supplementary material for: Polymers Used in US Household Cleaning Products: Assessment of Data Availability for Ecological Risk Assessment
Source: Integr Environ Assess Manag. 2019 Jul 26;15(4):621–32. doi: 10.1002/ieam.4150 (PMC6851760; doi:10.1002/ieam.4150)
Supplement: Supplementary file 1 — Supporting information [file IEAM-15-621-s001.docx]

**Authors:** Alison Pecquet†*, Drew McAvoy§, Charles Pittinger‡, Kathleen Stanton#

†Department of Environmental Health, University of Cincinnati, Cincinnati, OH, USA.

§ Department of Chemical and Environmental Engineering, University of Cincinnati, Cincinnati, OH, USA.

‡ Charles Pittinger LLC, Cincinnati, OH, USA.

# American Cleaning Institute, Washington, DC, USA.

**Supplemental Data File**

**S-1. Regulatory and NGO Definitions of Polymers**

Governmental definitions of polymers presented here were largely synthesized from a commissioned report: *Polymer Regulations and Polymer Notifications Update Report 2011*, Prepared by CIRS Europe (<http://www.cirs-reach.com>). This source was supplemented by reviews of regulations in key countries. Below we list the various definition’s for polymers as well as polymers of low concern criteria by country. While the US and other member countries adopted the OECD definition in 1993, below we list the country-specific regulation and supplemental information applied by each country. For example, the US has adopted the OECD definition of a polymer, but has specific regulation on polymer exemptions and Exclusions from Exemption, as listed.

The 1990 OECD Definition Adopted Internationally in 1993

(<http://www.oecd.org/dataoecd/3/23/42081261.pdf>)

The polymer definition was developed by the OECD as a result of the Experts on Polymers Meetings held in Toronto, Canada (January 1990), Paris, France (October 1991), and Tokyo, Japan (April 1993). The definition was agreed upon in May 1993 by the OECD member countries, including the United States, Canada, China, Korea, Japan, and member nations of the European Union. The OECD definition is:

*A 'POLYMER' is a substance consisting of molecules characterized by the sequence of one or more types of monomer units and comprising a simple weight majority of molecules containing at least three monomer units which are covalently bound to at least one other monomer unit or other reactant and consists of less than a simple weight majority of molecules of the same molecular weight. Such molecules must be distributed over a range of molecular weights wherein differences in the molecular weight are primarily attributable to differences in the number of monomer units.*

For deciding if a substance meets the definition of a polymer, these sequence and distribution criteria must be met:

• > 50 percent of molecules must be composed of a sequence of at least 3 monomer units plus at least one additional monomer unit or other reactant. (In other words, > 50 percent of the substance must be polymer molecules.)

• The amount of polymer molecules of any one molecular weight cannot exceed 50 weight percent.

By this definition, many low molecular weight substances, oligomeric reaction products, dimers or trimers would not be classified as a polymer.

The OECD preferred analytical method for determining whether a substance falls under the definition of a polymer is Gel Permeation Chromatography (GPC). Guidelines on the determination of the number average molecular weight (MWn) and molecular weight distribution using GPC are available in the OECD TG 118 (1996).

United States Definition and Management of Polymers

EPA website: Document18 <https://www.epa.gov/sites/production/files/2015-03/documents/polyguid.pdf>

The U.S. EPA, under the jurisdiction of the Toxic Substances Control Act, has regulated the use of polymers since the inception of Premanufacture Notifications (PMNs) in 1979. EPA’s definition and treatment of polymers evolved in the 1980’s. In 1993 the U.S. adopted the OECD’s 1990 definition above.

Relevant to this project, EPA has issued polymer classification criteria in two independent programs. In a non-enforceable guidance document, there are quantitative criteria on polymers, in: Interpretive Assistance Document for Polymers for Sustainable Futures Screening Assessments (EPA 2013). They distinguish and classify three types of low-concern polymers based on by Average Number Molecular Weight (MWn) and Low Molecular Weight (LMW) Material Composition.

The TSCA Polymer Exemption Rule.

This Rule is highly relevant and useful to the project, because it is most prescriptive and technical polymer exemption/PLC guidance internationally. It presents a broad range of technical criteria that EPA has considered in polymer classification and exemption. It also proscribes classes of polymers, some relevant to the cleaning industry (e.g., cationic polymers), that cannot be exempted from the full, new chemicals review process.

*Background.* In 1984, the EPA identified certain criteria to determine which polymers were most unlikely to present an unreasonable risk of injury to human health or the environment and could then be exempted from the onerous process of registration under the Premanufacture Notification requirements. The original polymer exemption rule under §5(h)(4) in 1984, revised in allowed polymers that met certain criteria under these conservative guidelines to be exempt from some of the reporting requirements for new chemicals (USEPA 1984). The polymer exemption rule was a precursor to the concept of the “Polymer of Low Concern”.

The current (1993) polymer exemption rule allows certain polymers to be exempt from the reporting requirements for new chemicals and imposes new restrictions on a limited set of polymers that were previously eligible for the exemption.

*Criteria.* To be eligible for the exemption, a new chemical substance must: 1) meet the polymer definition, 2) meet one of three exemption criteria, and 3) not be excluded. In addition to meeting the OECD polymer definition, a polymer must meet the following criteria.

1. If the average molecular weight (MW) of the polymer is between 1,000 - 10,000 Da, then the weight percentage of oligomer with MW < 500 must be less than 10 percent, and the weight percentage of oligomer with MW < 1000 must be less than 25 percent;
2. If the average molecular weight (MW) of the polymer is greater than 10,000 Da, then the weight percentage of oligomer with MW < 500 must be less than 2 percent and the weight percentage of oligomer with MW < 1000 must be less than 5 percent; and
3. Polyester polymer must be manufactured from a list of acceptable reactants.

Exclusions from Exemption

In addition to meeting the specific criteria of one of the three exemption types described above, a new polymer must not fall into any of the prohibited categories listed at §723.250(d) of the new rule. If it does, it is subject to full PMN reporting requirements.

This section of the amended rule specifically excludes certain polymers from the reduced reporting requirements of the polymer exemption. Some of the following exclusions are relevant to the cleaning industry.

- *Positively charged polymers.* Cationic or potentially cationic polymer cannot be exempt unless its charge density is sufficiently low or it is a non-dispersible, non-soluble solid;
- *Atomic element limitations.* All eligible polymers must contain as an integral part of their composition two or more of the atomic elements carbon, hydrogen, nitrogen, oxygen, silicon, and sulfur;
- *Instability.* Polymers that substantially degrade, decompose, or depolymerize are not eligible for exemption;
- *Un-reviewed reactants (2% rule).* Polymers that contain > 2% w/w of reactants or monomers that are not listed on the TSCA inventory are not eligible for exemption;
- *High molecular weight, water-absorbing polymers.* Polymers that are capable of absorbing its own weight of water and have a number-average molecular weight > 10,000 are not eligible for exemption; and
- *Reactive functional group.*

Cationic polymers and those polymers which are reasonably anticipated to become cationic in the natural aquatic environment are excluded from the exemption and may not be manufactured under it. The principal concern is the toxicity toward aquatic organisms. Further criteria for cationic polymers and other exclusions are described in USEPA’s 1997 Polymer Exemption Guidance Manual.

These exclusions may be relevant to certain cleaning industry polymers. We recommend that these criteria be considered in the polymer database to be created.

Polymer Definition under REACH / ECHA

From: ECHA Guidance on Polymers and Monomers 2012.

https://echa.europa.eu/documents/10162/23036412/polymers_en.pdf

*“A polymer is a substance consisting of molecules characterized by the sequence of one or more types of monomer units. Such molecules must be distributed over a range of molecular weights. Differences in the molecular weight are primarily attributable to differences in the number of monomer units.”*

In accordance with REACH (Article 3(5)), a polymer is defined as a substance meeting the following criteria:

*(a) Over 50 percent of the weight for that substance consists of polymer molecules (see definition below); and*

*(b) The amount of polymer molecules presenting the same molecular weight must be less than 50 weight percent of the substance.*

In the context of this definition:

- *A "polymer molecule" is a molecule that contains a sequence of at least 3 monomer units, which are covalently bound to at least one other monomer unit or other reactant.*
- *A "monomer unit" is the reacted form of a monomer substance in a polymer. For the identification of the monomeric unit(s) in the chemical structure of the polymer the mechanism of polymer formation may, for instance, be taken into consideration.*
- *A "sequence" is a continuous string of monomer units within the molecule that are covalently bonded to one another and are uninterrupted by units other than monomer units.*

Monomer Definition (also from ECHA)

*REACH defines a monomer as a substance which is capable of forming covalent bonds with a sequence of additional like or unlike molecules under the conditions of the relevant polymer-forming reaction used for the particular process (Article 3(6)). In other words, it is a substance which, via the polymerization reaction, is converted into a repeating unit of the polymer sequence.*

Substances exclusively involved in the catalysis, initiation or termination of the polymer reaction are not considered monomers. Any monomer is therefore by definition an intermediate. Nonetheless, the specific provisions for the registration of intermediates under REACH do not apply to monomers.

Canada’s Definition

From the Guidelines for the Notification and Testing of New Substances: Chemicals and Polymers, Pursuant to Section 69 of the *Canadian Environmental Protection Act, 1999*. — Version 2005. Co-published by: Environment Canada and Health Canada. [www.ec.gc.ca/substances/](http://www.ec.gc.ca/substances/)

Canada has adopted the OECD definition and criteria. Polymers are defined in subsection 1(1) of the Regulations as substances that consist of:

*(1) Molecules characterized by the sequence of one or more types of monomer units;*

*(2) With greater than 50% by weight of the molecules having three of more monomer units which are covalently bound to one or more other monomer units or reactants;*

*(3) With less than 50% by weight of the molecules having the same molecular weight; and*

*(4) With the molecules distributed over a range of molecular weights whose differences in molecular weights are primarily attributable to differences in the number of monomer units.*

For biopolymers the monomer units and reactants are considered to be the repeating units within the polymeric substance, which are either produced *in situ* by the organism or added to the reaction vessel. An example of a biopolymer is the polysaccharide xanthan gum, produced by *Xanthomonas camprestris*.

Note that polymers derived from a whole plant, an animal, or from parts of a whole plant or animal are not biopolymers for the purpose of the Regulations and must be notified as polymers.

China’s Definition

(*See CIRS 2011*).

China has adopted the OECD definition of a polymer and applies the same three criteria. Similarly, the preferred method in China to determine whether a substance falls under the definition of a polymer is Gel Permeation Chromatography (GPC).

China has also adopted the concept of a Polymer of Low Concern (PLC) proposed by OECD, even though China is not a member of OECD. China also adopted the same criteria as USEPA criteria under the TSCA Polymer Exemption Rule.

In China, a polymer that meets any one of the following three criteria will be regarded as a PLC.

1. If the average molecular weight (MW) of the polymer is between 1,000 - 10,000 Da, and at the same time, the weight percentage of oligomer with MW < 500 is less than 10 percent and the weight percentage of oligomer with MW < 1000 is less than 25 percent. Also, the polymer shall not contain functional groups of high concern (for example, heavy metals) and highly reactive functional groups.
2. If the average molecular weight (MW) of the polymer is greater than10,000 Da, and at the same time, the weight percentage of oligomer with MW < 500 is less than 2 percent and the weight percentage of oligomer with MW < 1000 is less than 5 percent.
3. If it is a Polyester polymer.

Personal Care Products Council (PCPC) Definition

While the focus of the PCPC is on plastic materials, their definition of polymeric substances has been agreed to by their company experts. Their definition is:

*Polymer is defined as a substance consisting of molecules characterized by the sequence of one or more types of monomer units and comprising a simple weight majority of molecules containing at least three monomer units which are covalently bound to at least one other monomer unit or other reactants and consists of less than a simple weight majority of molecules of the same molecular weight. Such molecules must be distributed over a range of molecular weights where differences in the molecular weights are primarily attributable in the number of monomer units. In the context of this definition a monomer unit means the reacted form of a monomer in a polymer.*

**References:**

[CIRS] Chemical Inspection and Regulation Services. 2011. Polymer Regulations and Polymer Notifications Update Report. Prepared by Queenier Yang and Edwin Wen, CIRS Global Chemical Regulations R&D Centre. [cited 2018 March 27]. <http://www.cirs-reach.com/Polymer_Regulations_New_Polymer_Notificatios_Update_Report.pdf>

[ECHA] European Chemicals Agency. 2012. Guidance on Monomers and Polymers. Version 2.0 Guidance for the implementation of REACH. <https://echa.europa.eu/documents/10162/23036412/polymers_en.pdf>

[EFSA] European Food Safety Authority. 2008. Note for Guidance on Food Contact Materials. [cited 2018 March 27]. <http://www.foodcontactmaterials.com/eu/noteforguidance.pdf>

[HC] Health Canada. 2005 Guidelines for the Notification and Testing of New Substances: Chemicals and Polymers, Pursuant to Section 69 of the Canadian Environmental Protection Act, 1999. Version 2005. ISBN 0-662-69285-3 Cat. no.: En84-25/2005. [cited 2018 March 27]. <http://www.ec.gc.ca/ese-ees/02C6D66D-A211-E2B8-74C0-01D17A6D6FC0/cpguidem688.pdf>

[OECD] Organisation for Economic Cooperation and Development. 1990. OECD Definition of Polymer. [cited 2018 March 27]. <http://www.oecd.org/env/ehs/oecddefinitionofpolymer.htm>

[OECD] Organisation for Economic Cooperation and Development. 1993. OECD Expert Group on Polymers. Third Meeting of the Experts on Polymers: Chairman’s Report [ENV/MC/CHEM/RD(93)4], April 1993, Paris, France.

[OECD] Organisation for Economic Cooperation and Development. 2009. Data analysis of the identification of correlations between polymer characteristics and potential for health or ecotoxicological concern. Joint Meeting of the Chemicals Committee and the Working Party on Chemicals, Pesticides, and Biotechnology, Environment Directorate. ENV/JM/MONO(2009)1. [cited 2018 March 27]. <https://www.oecd.org/env/ehs/risk-assessment/42081261.pdf>

[PCPC] Personal Care Products Council. 2016. International Plastic Definition Workshop Overview. Unpublished Report.

[US EPA] United States Environmental Protection Agency. 1997. Polymer Exemption Guidance Manual. Office of Pollution Prevention and Toxics. June 1997, EPA744-B-97-001.

**S-2. Published literature and database searching**

The aim of this task was to identify published and unpublished data that can be used to conduct an ecological risk assessment. The focus is on identifying data gaps and prioritizing compounds for future risk assessment. A systematic literature search was conducted to identify currently available environmental safety data for the polymers identified in the final list.

We employed a multipronged approach that included a comprehensive literature search of open-access regulatory and related databases, and published journal literature with tree searching. In addition, data from authoritative sources such as reference books (e.g., Ecological Assessment of Polymers) and Internet searches were included. This data identification strategy was used in all phases of the research related to environmental safety endpoints.

*Published literature searching*

We conducted comprehensive and systematic literature searches utilizing four common databases. The databases searched were *Scopus*, *Web of Science*, *Environment Complete* (including *Academic Search* *Complete*, *Academic Search Premier*, and *Agricola*), and *Toxline* (including *PubMed* results). These databases were searched first using the CAS RN. If there were no hits, the chemical name as listed in the polymer inventory spreadsheet was used. If either of these searches produced over 100 hits, key terminology using the AND function during searching were used to help limit the results to only those in scope of this project. These terms varied slightly depending on the specific database being search, but included: toxic*, eco*, ecolog*, ecotox*, environ*, risk, and risk assessment, or variations thereof.

Since the polymers of interest are grouped categorically, additional chemical category searches were conducted in a similar manner, utilizing AND functions when needed.

Once these studies were determined to be potentially relevant, they were downloaded as titles and abstracts from each database into a large EndNote library. In this library, studies were again grouped by chemical category in an attempt to minimize the number of duplicate studies across polymers. The total number of studies identified was 731 (*Table S-1*). After initial screening for relevance, duplicates, and for specific chemical data, the refined database contained 457 potentially relevant studies. These abstracts and titles were then reviewed by our senior scientists for relevance.

Once an abstract was deemed relevant or potentially relevant to this research, the full manuscript was retrieved to determine if there were any relevant data that could be used in a risk assessment. After this review by our senior scientists, a total of 35 studies (from the initial screen of 457) were retained (*Table S-1*).

**Table S-1. Total results of literature searches per chemical category across all databases.**

| **Chemical class** | **Total number of retained hits*** | **After removal of non-relevant and duplicates** | **After final review for relevant data** |
| --- | --- | --- | --- |
| Carbohydrates | 88 | 44 | 14 |
| Ethers | 140 | 92 | 4 |
| Glycerides | 28 | 19 | 5 |
| Polybutenes | 53 | 25 | 1 |
| Polycarboxylates | 108 | 69 | 0^#^ |
| Polyethyleneimine | 2 | 2 | 1 |
| Polymer | 37 | 25 | 0 |
| Polyols | 75 | 55 | 6 |
| Polystyrene | 65 | 47 | 0 |
| Quaternary ammonium compounds | 104 | 60 | 2 |
| Resins | 13 | 12 | 2 |
| Sulfosuccinates/sulfosuccimates | 18 | 7 | 0 |
| **TOTAL** | **731** | **457** | **35** |

*Does not account for duplicates; ^#^ Not reviewed due to HERA reports

*Regulatory agency and additional database searching*

We utilized documentation of environmental effects and physical/chemical information from U.S., international agencies, and from additional data sources. Most government or regulatory agencies only provide chemical class-specific reports (no single chemical reports). Also, many of these general chemical category-specific reports were identified in the initial round of literature searching conducted during the scoping phase of this work.

The US EPA ECOTOX (<https://cfpub.epa.gov/ecotox/quick_query.htm>) database was searched using the chemical CAS for safety data on the polymers of interest. Overall, this database provided 191 terrestrial data points and 191 aquatic data points. These data were incorporated into the data gap identification, after a vetting for their usefulness and applicability was performed.

International databases were also searched for data specific to the polymers of interest. An in-depth search for international regulatory agencies was not conducted because it was felt that the data already identified in the US EPA ECOTOX database captured most of the available data, and the tendency of regulatory agencies to focus on categorical reports as opposed to chemical-specific reports. However, the ECHA database (<https://echa.europa.eu/information-on-chemicals>) was searched by CAS for the polymers of interest, as this was anticipated to capture any data submissions under REACH.

In the ECHA database, there were eight polymers with dossier submissions. These data were incorporated into the data gap identification, after a vetting for their usefulness and applicability.

The US EPA Safer Choice Chemical Ingredient List (<https://www.epa.gov/saferchoice/safer-ingredients>) was also searched to see if any of the polymers of interest were listed. Thirty-two polymers were on this list and coded as “green circles”, which means the “chemical has been verified to be of low concern based on experimental and modeled data”. Additionally two polymers were on the “green half-circle” list, which means the “chemical is expected to be of low concern based on experimental and modeled data, and one polymer was on the “yellow triangle” list, which means the “chemical has met the Safer Choice Criteria for its functional ingredient class, but it has some hazard profile issues”. It should be noted that the data used in these assessments are not provided on the US EPA web site, only the final assessment outcome.

**S-3. Literature searches for the 18 polymers**

**Table B1. Search results for chemicals in the Environment complete (EBSCO) Database including: academic search complete, academic search premier, and Agricola.**

| **CAS RN** | **Polymer Name** | **Search string** | **hits** | **saved for review#** |
| --- | --- | --- | --- | --- |
| 25133-97-5 | Acrylate Copolymer | 25133-97-5 Acrylate Copolymer Acrylate Copolymer AND ecolog* Acrylate Copolymer AND toxicol* | 0 2310 5 4 | 0 refined 1 2 |
| 30351-73-6 | P(AA/EA/MAA) [polymers of acrylic acid, ethyl acrylate, methyl acrylic acid] | 30351-73-6 polymers of acrylic acid, ethyl acrylate, methyl acrylic acid | 0 1 | 0 0 |
| 25034-86-0 | Styrene/Acrylates Copolymer | 25034-86-0 | 0 | 0 |
| 25085-34-1 | Styrene/Acrylates Copolymer | 25085-34-1 | 0 | 0 |
| 40623-75-4 | AA/AMPS copolymer | 40623-75-4  Acrylate/sulfonate copolymer AA/AMPS copolymer | 0  0  6 | 0  0  0 |
| 9011-13-6 | Styrene/MA Copolymer | 9011-13-6 Styrene methacrylate Copolymer AND ecolog* Styrene methacrylate Copolymer AND ecotox* | 0 4 0 | 0 0 0 |
| 24981-13-3 | Styrene/acrylamide copolymer | 24981-13-3 | 0 | 0 |
| 68130-99-4 | Polyethyleneimine Ethoxylate | 68130-99-4 Polyethyleneimine Ethoxylate | 0 0 | 0 0 |
| 54590-72-6 | Polyester-5 | 54590-72-6 Polyester-5 | 0 6 | 0 0 |
| 9007-48-1 | Polyglyceryl Oleate | 9007-48-1 Polyglyceryl Oleate | 0 4 | 0 0 |
| 3055-97-8 | Alcohols, Lauryl, Ethoxylated | 3055-97-8 Alcohols, Lauryl, Ethoxylated ether AND ecotoxic* ether polymer AND ecolog* | 0 5 885 6 | 0 0 reviewed first 90, saved 6 1 |
| 26006-22-4 | Polyquaternium 5 | 26006-22-4 Polyquaternium 5 Polyquaternium AND ecolog* Polyquaternium AND ecotox* | 0 0 1 1 | 0 0 0 1 |
| 26062-79-3 | Polyquaternium 6 | 26062-79-3 Polyquaternium 6 | 0 2 | 0 0 |
| 26590-05-6 | Polyquaternium 7 | 26590-05-6 Polyquaternium 7 | 0 6 | 0 0 |
| 68555-36-2 | Polyquaternium 2 | 68555-36-2 Polyquaternium 2 | 0 8 | 0 0 |
| 68610-92-4 | Polyquaternium 10 | 68610-92-4 Polyquaternium 10 | 0 17 | 0 1 |
| 9003-08-1 | Melamine resin | 9003-08-1 Melamine resin Melamine resin AND toxicol* Melamine resin AND ecolog* resin polymers AND ecolog* | 0 1023 3 4 15 | 0 refined 0 0 3 |
| 52906-93-1 | Starch, hydrogen 2-(octen-1-yl) butanedioate; Butanedioate | 52906-93-1 | 0 | 0 |

**Table B2. Search results for chemicals in the Web of Science: Core collection.**

| **CAS RN** | **Polymer Name** | **Search string** | **hits** | **saved for review#** |
| --- | --- | --- | --- | --- |
| 25133-97-5 | Acrylate Copolymer | 25133-97-5 Acrylate Copolymer Acrylate Copolymer AND ecolog* Acrylate Copolymer AND toxicol* | 0 11,160 6 5 | 0 refined 0 3 |
| 30351-73-6 | P(AA/EA/MAA) [polymers of acrylic acid, ethyl acrylate, methyl acrylic acid] | 30351-73-6 polymers of acrylic acid, ethyl acrylate, methyl acrylic acid (polymers of acrylic acid, ethyl acrylate, methyl acrylic acid) AND eco* (polymers of acrylic acid, ethyl acrylate, methyl acrylic acid) AND toxic* | 0 0  3 | 0 0  2 |
| 25034-86-0 | Styrene/Acrylates Copolymer | 25034-86-0 | 0 | 0 |
| 25085-34-1 | Styrene/Acrylates Copolymer | 25085-34-1 | 0 | 0 |
| 40623-75-4 | AA/AMPS copolymer | 40623-75-4  Acrylate/sulfonate copolymer AA/AMPS copolymer | 0  3  22 | 0  0  0 |
| 9011-13-6 | Styrene/MA Copolymer | 9011-13-6 Styrene methacrylate Copolymer AND ecolog* Styrene methacrylate Copolymer AND ecotox* | 0 0 0 | 0 0 0 |
| 24981-13-3 | Styrene/acrylamide copolymer | 24981-13-3 | 0 | 0 |
| 68130-99-4 | Polyethyleneimine Ethoxylate | 68130-99-4 Polyethyleneimine Ethoxylate | 0 0 | 0 0 |
| 54590-72-6 | Polyester-5 | 54590-72-6 Polyester-5 | 0 17 | 0 0 |
| 9007-48-1 | Polyglyceryl Oleate | 9007-48-1 Polyglyceryl Oleate | 0 4 | 0 0 |
| 3055-97-8 | Alcohols, Lauryl, Ethoxylated | 3055-97-8 Alcohols, Lauryl, Ethoxylated ether AND ecotoxic* ether polymer AND ecolog* | 0 24 139 14 | 0 1 refined 2 |
| 26006-22-4 | Polyquaternium 5 | 26006-22-4 Polyquaternium AND ecotox* Polyquaternium 5 | 0 2 41 | 0 1 1 |
| 26062-79-3 | Polyquaternium 6 | 26062-79-3 | 0 38 | 0 0 |
| 26590-05-6 | Polyquaternium 7 | 26590-05-6 | 0 33 | 0 0 |
| 68555-36-2 | Polyquaternium 2 | 68555-36-2 Polyquaternium AND eco* | 0 2 | 0 0 |
| 68610-92-4 | Polyquaternium 10 | 68610-92-4 Polyquaternium 10 AND eco* | 0 1 | 0 1 |
| 9003-08-1 | Melamine resin | 9003-08-1 Melamine resin Melamine resin AND toxicol* Melamine resin AND ecolog* resin polymers AND ecotox* | 0 1851 4 5 3 | 0 refined 3 0 2 |
| 52906-93-1 | Starch, hydrogen 2-(octen-1-yl) butanedioate; Butanedioate | 52906-93-1 | 0 | 0 |

**Table B3. Search results for chemicals in the Scopus Database: Core collection.**

| **CAS RN** | **Polymer Name** | **Search string** | **hits** | **saved for review#** |
| --- | --- | --- | --- | --- |
| 25133-97-5 | Acrylate Copolymer | 25133-97-5 Acrylate Copolymer Acrylate Copolymer AND ecolog* Acrylate Copolymer AND ecotox* (acrylate copolymer AND biodeg* AND (eco* OR environ*)) | 0 10,921 8 1 23 | 0 refined 0 0 2 |
| 30351-73-6 | P(AA/EA/MAA) [polymers of acrylic acid, ethyl acrylate, methyl acrylic acid] | 30351-73-6 polymers of acrylic acid, ethyl acrylate, methyl acrylic acid (polymers of acrylic acid, ethyl acrylate, methyl acrylic acid) AND eco* (polymers of acrylic acid, ethyl acrylate, methyl acrylic acid) AND toxic* | 0 264 0  4 | 0 refined 0  1 |
| 25034-86-0 | Styrene/Acrylates Copolymer | 25034-86-0 | 0 | 0 |
| 25085-34-1 | Styrene/Acrylates Copolymer | 25085-34-1 | 0 | 0 |
| 40623-75-4 | AA/AMPS copolymer | 40623-75-4  Acrylate/sulfonate copolymer  Acrylate/sulfonate copolymer AND eco*  Acrylate/sulfonate copolymer AND toxic*  AA/AMPS copolymer  AA/AMPS copolymer AND eco*  AA/AMPS copolymer AND toxic* | 0  159  1  1  126  4  3 | 0  refined  0  0  refined  0  0 |
| 9011-13-6 | Styrene/MA Copolymer | 9011-13-6 Styrene methacrylate Copolymer AND ecolog* Styrene methacrylate Copolymer AND ecotox* Styrene methacrylate Copolymer AND biodeg* | 0 3 0 36 | 0 0 0 0 |
| 24981-13-3 | Styrene/acrylamide copolymer | 24981-13-3 | 0 | 0 |
| 68130-99-4 | Polyethyleneimine Ethoxylate | 68130-99-4 Polyethyleneimine Ethoxylate | 0 1 | 0 0 |
| 54590-72-6 | Polyester-5 | 54590-72-6 Polyester-5 | 0 17 | 0 1 |
| 9007-48-1 | Polyglyceryl Oleate | 9007-48-1 Polyglyceryl Oleate | 0 15 | 0 0 |
| 3055-97-8 | Alcohols, Lauryl, Ethoxylated | 3055-97-8 Alcohols, Lauryl, Ethoxylated ether AND ecotoxic* ether polymer AND ecolog*  ether AND ecolog* OR ecotox* AND risk) AND NOT (diphenyl) AND NOT (bde OR pbde)) | 0 40 366 669 72 | 0 8 refined 2 13 |
| 26006-22-4 | Polyquaternium 5 | 26006-22-4 Polyquaternium AND ecotox* Polyquaternium AND ecolog* "Polyquaternium 5" | 0 1 0 0 | 0 1 0 1 |
| 26062-79-3 | Polyquaternium 6 | 26062-79-3 "Polyquaternium 6" | 0 10 | 0 0 |
| 26590-05-6 | Polyquaternium 7 | 26590-05-6 "Polyquaternium 7" | 0 13 | 0 2 |
| 68555-36-2 | Polyquaternium 2 | 68555-36-2 Polyquaternium AND eco* | 0 3 | 0 1 |
| 68610-92-4 | Polyquaternium 10 | 68610-92-4 Polyquaternium 10 AND eco* | 0 1 | 0 1 |
| 9003-08-1 | Melamine resin | 9003-08-1 Melamine resin Melamine resin AND ecolog* Melamine resin AND ecotox* resin polymers AND ecotox* melamine resin polymers AND biodeg* | 0 3434 20 1 21 6 | 0 refined 0 0 5 0 |
| 52906-93-1 | Starch, hydrogen 2-(octen-1-yl) butanedioate; Butanedioate | 52906-93-1 | 0 | 0 |

**Table B4. Search results for chemicals in the ToxLine Database: Core collection.**

| **CAS RN** | **Polymer Name** | **Alternative Chemical Classification** | **Original Chemical Classification** | **Search string** | **hits** | **saved for review#** |
| --- | --- | --- | --- | --- | --- | --- |
| 25133-97-5 | Acrylate Copolymer | Polycarboxylate - acrylate copolymer | Polycarboxylates | 25133-97-5 (Acrylate Copolymer) AND (biodeg* OR ecotox* OR ecolog*) | 0 17 | 0 5 |
| 30351-73-6 | P(AA/EA/MAA) [polymers of acrylic acid, ethyl acrylate, methyl acrylic acid] | polycarboxylate | rheology modifier | 30351-73-6 OR polymers of acrylic acid, ethyl acrylate, methyl acrylic acid | 6 | 2 |
| 25034-86-0 | Styrene/Acrylates Copolymer | styrene and Vinyl-type Styrene Copolymers | Polycarboxylates | 25034-86-0 | 4 | 0 |
| 25085-34-1 | Styrene/Acrylates Copolymer | styrene and Vinyl-type Styrene Copolymers | Polycarboxylates | 25085-34-1 | 1 | 1 |
| 40623-75-4 | AA/AMPS copolymer | Acrylate/sulfonate copolymer | Acrylate/sulfonate copolymer | 40623-75-4  Acrylate/sulfonate copolymer  AA/AMPS copolymer | 1  3  3 | 0  0  0 |
| 9011-13-6 | Styrene/MA Copolymer | styrene and Vinyl-type Styrene Copolymers |  | (9011-13-6 OR Styrene methacrylate Copolymer) AND (biodeg* OR ecotox* OR ecolog*) | 4 | 1 |
| 24981-13-3 | Styrene/acrylamide copolymer | styrene and Vinyl-type Styrene Copolymers |  | 24981-13-3 | 2 | 0 |
| 68130-99-4 | Polyethyleneimine Ethoxylate | Polyethyleneimine and derivatives (polyelectrolytes) | Polyethyleneimine and Derivatives | 68130-99-4 OR Polyethyleneimine Ethoxylate | 5 | 0 |
| 54590-72-6 | Polyester-5 |  | Polymer | (54590-72-6 OR Polyester-5) AND (ecotox* OR ecolog*) | 26 | 2 |
| 9007-48-1 | Polyglyceryl Oleate | polyglycerol esters | [grouped with glycerols] | 9007-48-1 OR Polyglyceryl Oleate | 1 | 0 |
| 3055-97-8 | Alcohols, Lauryl, Ethoxylated | Ethers | Ethers | 3055-97-8 Alcohols, Lauryl, Ethoxylated ether AND ecotoxic* ether polymer AND ecotoxic*  ether polymer AND ecolog* | 2 5 332 5 8 | 0 2 refined 0 2 |
| 26006-22-4 | Polyquaternium 5 | Quaternary Ammonium Polymer | Quaternary Ammonium Compounds | 26006-22-4 (Polyquaternium OR "Polyquaternium 5") AND (ecotox* OR ecolog*) | 1 3 | 1 1 |
| 26062-79-3 | Polyquaternium 6 | Quaternary Ammonium Polymer | Quaternary Ammonium Compounds | (26062-79-3 OR "Polyquaternium 6") AND (ecotox* OR ecolog*) | 6 | 5 |
| 26590-05-6 | Polyquaternium 7 | Quaternary Ammonium Polymer | Quaternary Ammonium Compounds | (26590-05-6 "Polyquaternium 7") | 1 | 1 |
| 68555-36-2 | Polyquaternium 2 | Quaternary Ammonium Polymer | Quaternary Ammonium Compounds | (68555-36-2 OR Polyquaternium) AND (ecotox* or ecolog*) | 3 | 1 |
| 68610-92-4 | Polyquaternium 10 | Quaternary Ammonium Polymer | Quaternary Ammonium Compounds | (68610-92-4 OR "Polyquaternium 10") AND (ecotox* or ecolog*) | 2 | 2 |
| 9003-08-1 | Melamine resin | resin |  | (9003-08-1 OR Melamine resin) AND (ecotox* OR ecolog* OR biodeg*) | 8 | 2 |
| 52906-93-1 | Starch, hydrogen 2-(octen-1-yl) butanedioate; Butanedioate | Carbohydrates | Carbohydrates | 52906-93-1 | 2 | 0 |
